# Exogenous Glycinebetaine Regulates the Contrasting Responses in Leaf Physiochemical Attributes and Growth of Maize under Drought and Flooding Stresses

**DOI:** 10.3390/biology13060360

**Published:** 2024-05-21

**Authors:** Guo-Yun Wang, Shakeel Ahmad, Bing-Wei Wang, Li-Bo Shi, Yong Wang, Cheng-Qiao Shi, Xun-Bo Zhou

**Affiliations:** 1 Guangxi Key Laboratory of Agro-Environment and Agro-Products Safety, Key Laboratory of Crop Cultivation and Physiology, College of Agriculture, Guangxi University, Nanning 530004, China; yuanyiwanggy@yeah.net (G.-Y.W.); shakeel@gxu.edu.cn (S.A.); wy5508@163.com (Y.W.); 2 Maize Research Institute, Guangxi Academy of Agricultural Sciences, Nanning 530007, China; sfsh515@163.com; 3 MAP Division (Shandong) of Sinochem Agriculture Holdings, Jinan 250000, China; shilibo689@163.com

**Keywords:** water stress, foliar glycinebetaine, antioxidant defense, reactive oxygen species, maize

## Abstract

**Simple Summary:**

Maize production is largely limited by flooding and drought. To mitigate the negative effects of abiotic stress, glycinebetaine is used in this study. However, there are not many research studies that focus on the different roles of glycinebetaine in resisting flooding and drought stresses. The objectives of this study were to reveal the diverse defense mechanisms to flooding and drought that are regulated by foliar glycinebetaine. Drought induced higher antioxidant activities and proline content compared to flooding, while flooding decreased superoxide dismutase as well as guaiacol peroxidase activities and proline content. Antioxidant ability and maize production were further enhanced by glycinebetaine. Glycinebetaine displayed more effects on leaves’ physiochemistry under flooding than drought, and it had a greater effect on the sensitive rather than the tolerant variety of maize. Overall, 5.0 mmol/L of glycinebetaine was determined to be the most optimal and effective concentration for mitigating the damage of water stress regarding maize production whilst also having a non-toxic effect. This study provides a potential strategy for mitigating the damage of water stress and for improving maize production.

**Abstract:**

Flooding and drought are the two most devastating natural hazards limiting maize production. Exogenous glycinebetaine (GB), an osmotic adjustment agent, has been extensively used but there is limited research on its role in mitigating the negative effects of different abiotic stresses. This study aims to identify the different roles of GB in regulating the diverse defense regulation of maize against drought and flooding. Hybrids of Yindieyu 9 and Heyu 397 grown in pots in a ventilated greenhouse were subjected to flooding (2–3 cm standing layer) and drought (40–45% field capacity) at the three-leaf stage for 8 d. The effects of different concentrations of foliar GB (0, 0.5, 1.0, 5.0, and 10.0 mM) on the physiochemical attributes and growth of maize were tested. Greater drought than flooding tolerance in both varieties to combat oxidative stress was associated with higher antioxidant activities and proline content. While flooding decreased superoxide dismutase and guaiacol peroxidase (POD) activities and proline content compared to normal water, they all declined with stress duration, leading to a larger reactive oxygen species compared to drought. It was POD under drought stress and ascorbate peroxidase under flooding stress that played crucial roles in tolerating water stress. Foliar GB further enhanced antioxidant ability and contributed more effects to POD to eliminate more hydrogen peroxide than the superoxide anion, promoting growth, especially for leaves under water stress. Furthermore, exogenous GB made a greater increment in Heyu 397 than Yindieyu 9, as well as flooding compared to drought. Overall, a GB concentration of 5.0 mM, with a non-toxic effect on well-watered maize, was determined to be optimal for the effective mitigation of water-stress damage to the physiochemical characteristics and growth of maize.

## 1. Introduction

China was recently ranked among the top 10 countries most severely impacted by frequent and alternating drought and flooding, leading to immense economic losses (EM-DAT2020-2022) [1]. As per EM-DAT2022, the notable drought and flooding events resulted in a total economic loss of US$ 12.6 billion for China in 2022. The simultaneous occurrence of drought and flooding has increased with global climate change, at the same time, severely limiting crop growth and production in Guangxi [2,3]. Drought and flooding have immense inhibitory effects on plants, leading to a series of biochemical and growth alterations [4,5]. The most typical response of plants to water stress (WS) is the excessive accumulation of reactive oxygen species (ROS), which causes oxidative damage to plants, inhibiting their growth [6]. Undoubtedly, crop growth slows down under WS, which is reflected by the reduction in leaf area (LA), plant height, stem diameter, and yield [7,8]. To avoid or tolerate WS, crops will generate a series of certain physiological responses through self-regulation to adapt to stress. For instance, the activities of antioxidant enzymes, such as superoxide dismutase (SOD), guaiacol peroxidase (POD), and ascorbate peroxidase (APX), and the content of osmotic adjustment substances such as proline and soluble sugars, are enhanced to cope with oxidative stress damages [9,10]. However, there are considerable inconsistencies in the literature regarding the regulation employed by different crops’ responses to drought and flooding. Previous research has reported that flooding has no significant effect on LA but greatly increases stem dry matter, SOD, and POD activities [11,12,13]. On the contrary, other studies have shown that SOD, POD, and APX activities and LA drastically decreased under flooding stress [4,13]. Recently, a few studies have highlighted the distinct stress response and defense mechanism of maize (*Zea mays* L.), based on their physiological and biochemical response to drought and flooding [3,5,14,15]. However, it will be still necessary to clarify the antioxidant defense stress mechanism of physiochemical attributes and growth of maize when maize suffers from drought and flooding stresses at the same time.

Despite being a water-loving crop, maize is sensitive to WS [6,12]. The water sensitivity and tolerance of plants to WS vary with the growth stage and stress duration. For maize, the intensity of the adverse impacts from flooding generally improves with the lengthening of stress duration but declines from the three-leaf stage (V3) to the tasseling stage [4,16]. The most sensitive stage of maize to flooding is generated at the V3, in which leaves accumulate more hydrogen peroxide (H_2_O_2_) and superoxide anion (O_2_^−^) compared to the five-leaf stage (V5) [12]. Concurrently, flooding causes considerable reductions in the grain yield, dry matter content, and LA of maize [4,7,17]. While short-term WS generally limits crop growth, long-term WS damages plants to the point of no recovery [14,17,18]. Therefore, maize at the V3 should be used as a study object for understanding their defense and ROS scavenging mechanisms under WS.

To alleviate stress damage and improve stress tolerance in plants, exogenous application of growth regulators has been widely adopted [6,19,20,21]. Glycinebetaine (GB), as a quaternary amine compound, is an osmolyte, which mainly is distributed in chloroplasts and cytoplasm [22] The GB acts as an osmotic adjustment substance and helps in enhancing the tolerance of crops against abiotic stress by increasing the antioxidant ability and protecting the biological membranes from oxidative stress caused by excess ROS [19,23,24]. The GB has been widely applied in multiple plants, such as maize [25], wheat [26], tomato [27], and cotton [21], under various abiotic stresses because it is easily extracted and highly stable in plant tissues [24]. Although many studies have shown that GB accumulation is positively correlated with the stress tolerance of plants, the relationship will be changed with the variety, concentration, and application method [21,23,24]. Maize leaves have a high ability to absorb and accumulate exogenous GB and can immediately transfer GB from leaf to root [28], indicating that foliar application of GB will be an optimum strategy. The most effective and efficient concentration of GB for stress tolerance may vary with different plants and varieties [21,29]. The GB improves drought tolerance more for tolerant than sensitive varieties [23]. However, differences have been found in other studies, where GB has the same effect on various varieties [30,31]. The various responses of different stress-tolerant varieties caused by GB will need to be further clarified. A low dosage of GB when it is sprayed on leaves greatly promotes plant growth under stress or non-stress conditions; however, a high dosage can have an inhibitory but non-harmful effect on plants [21,32]. Yang and Lu [32] have reported that dry weight and plant height increase with a concentration of GB up to 10 mM, while are inhibited by GB > 10 mM. An optimum dosage of GB (5 mM) significantly increases the gas exchange parameter of cotton [21]. For maize, different concentrations of GB are optimum for the foliar spray in different studies, including 0.5 mM [33], 1.0 mM [34], and 10 mM [25], implying that the concentration of GB for maize varies with treatments and is limited for studies related to flooding. In addition, there are limited studies that focus on the contrasting regulation of GB on stress duration, as well as on both drought and flooding. Therefore, the optimum dosage of foliar GB needs to be determined for maize response to drought and flooding stresses.

Further research is still needed to fully understand the numerous defense regulations and stress tolerance of plants to drought and flooding, as well as the possible involvement of exogenous GB in the defense process. Therefore, our main objectives in this study were to (1) detect the different stress defenses and ROS scavenging mechanisms of maize leaves when separately subjected to drought and flooding; (2) determine the optimum concentration of GB for foliar spray on maize leaves at V3; and (3) determine the different effects of GB on water-tolerant or -sensitive varieties of maize under drought and flooding. The results of this study will be crucial for promoting sustainable cultivation and management of maize.

## 2. Materials and Methods

### 2.1. Plant Materials and Experimental Design

The experiment was set up in a ventilated greenhouse at Guangxi University, an experimental station in Nanning, Guangxi, China (22°50′ N, 108°17′ E). The annual average of precipitation is 1599.0 mm, the temperature is 20.5 °C, and the relative humidity is 79.1% over a 30-year period in the region, and the prevailing climate is a humid subtropical monsoon climate.

**Figure 1:**
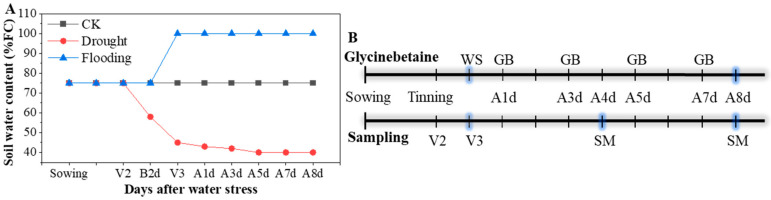
The change in soil water content (**A**) and the diagram of the time (**B**) for foliar glycinebetaine (GB) and sampling during the experiment. FC represents maximum field capacity; CK represents maize planted in normal field capacity; V2 and V3 are two-leaf stage and three-leaf stage for maize, respectively; B2d is 2 d before water stress (WS), and A1d, A3d, A4d, A5d, A7d, and A8d indicate 1, 3, 4, 5, 7, and 8 days after WS, respectively; SM is sampling and measuring for indicators.

Two maize (*Zea mays* L.) hybrids of Yindieyu 9 (tolerant to drought and flooding) and Heyu 397 (sensitive to drought and flooding) based on a previous study [3] were designed as the main plot and planted in plastic pots (32.5 cm diameter, and 29.0 cm height) on the 28th September 2020. The pots were arranged in a split-split plot design with three replications and placed at 60 cm row spacing and 32.5 cm center distance of the pot. Ten seeds were planted per pot, and five seedlings per pot were thinned at the two-leaf stage. During the early growth period of the maize seedlings, the soil water content was maintained at the normal field capacity (FC) of 70–75%. The seedlings at V3 were imposed with progressive drought (40–45% FC) and flooding (2.0–3.0 cm standing layer) stresses for 8 d; additionally, normal FC was set as a control (CK), and those were designed as the split-plot. The soil moisture content for CK and drought stress was controlled based on daily measurements of pot weight, in which each pot weight was controlled between 9.59 and 9.72 kg (70–75% FC) for CK and 8.88 and 9.00 kg for drought stress (40–45% FC); the details are shown in Figure 1A. For flooding stress, the water level was maintained using an additional big pot without holes placed in each pot. Different concentrations of GB with 0, 0.5, 1.0, 5.0, and 10.0 mM, designated hereafter as GB0, GB1, GB2, GB3, and GB4, respectively, were selected based on previous studies [21,25,33,34], designed as a split-split plot, and sprayed using a 500 mL watering pot to each pot with 25.0 mL after 1, 3, 5, and 7 days of stress (Figure 1B). After 4 d and 8 d of treatments, the indicators were measured, and all fully developed leaves per pot were sampled and stored at −80 °C until further analysis for ROS, antioxidant enzyme activities, and proline content (Figure 1B).

Each plastic pot was filled with 8.0 kg of dry soil (soil water content of 3.79%) from arable topsoil mixed with 4.49 g urea (46.2% N), 1.38 g muriate of potash (60.0% K_2_O), and 4.61 g calcium magnesium phosphate (18.0% P_2_O_5_). The arable topsoil was sandy clay loam (53.27% sand, 20.65% clay, and 26.28% silt, World Reference Base) with 31.21% FC (g/g, %), pH 6.83, soil bulk density of 1.24 g/cm^3^, soil organic matter of 24.14 g/kg, with available nitrogen of 0.12 g/kg, available phosphorus of 22.10 mg/kg, and available potassium of 69.50 mg/kg.

### 2.2. Sampling and Measurements

#### 2.2.1. Determination of Reactive Oxygen Species

The extraction method was performed according to Wang et al. [3]. Briefly, 0.2 g fresh leaf was crushed at 4 °C using a high-throughput cold grinding machine (Xinyi-48N, Ningbo Xinyi Ultrasonic Equipment Co., Ltd., Zhejiang, China). Then, a 2.0 mL pre-chilled phosphate buffer solution (PBS, 50 mM, pH 7.8) containing 1.0% polyvinyl pyrrolidone was added to the homogenate and fully oscillated. Subsequently, the homogenate was centrifuged at 12,000× *g* for 15 min at 4 °C, and the supernatant was ready to measure the O_2_^−^ content according to the slightly modified method of hydroxylamine oxidation [35]. The 0.5 mL of supernatant, sequentially mixed with 0.5 mL of 50 mM PBS (pH 7.8) and 1.0 mL of 10 mM hydroxylamine hydrochloride, was incubated at 25 °C for 1 h. After that, 1.0 mL of 17 mM para-aminobenzoic acid and 1.0 mL of 7 mM α-naphthylamine were added in that order to the reaction mixture. After 20 min of incubation at 25 °C, the absorbance of the mixture (OD) was measured at 540 nm using a luminometer (SpectraMax Plus384, Molecular Devices, CA, USA). The O_2_^−^ content was calculated according to Formula (1).

O_2_^−^ content (μg/g FW) = (*X* × *V_s_*)/(*FW* × *V_r_*) (1)

where FW is the fresh weight (g); *X* is the content of O_2_^−^ according to standard curve (μg); *V_s_* is the volume of sample extraction (mL), and *V_r_* is the volume of supernatant participating in the reaction (mL).

The H_2_O_2_ content was determined based on the slightly modified method of Velikova et al. [36]. A total of 0.2 g of ground fresh leaf, homogenized in 2.0 mL trichloroacetic acid (0.1%, *w*/*v*) homogenate, was centrifuged at 12,000× *g* for 15 min at 4 °C. After that, 0.5 mL of the supernatant was mixed with 0.5 mL of 10 mM PBS (pH 7.0) and 1 mL of 1.0 M potassium iodide. The OD_390_ was assayed using the described above luminometer and the content was calculated as follows:

H_2_O_2_ content (μM/g FW) = (*X* × *V_s_*)/(*FW* × *V_r_*) (2)

where *X* is the content of H_2_O_2_ according to the standard curve (μM); and others are the same as described in the O_2_^−^ content.

#### 2.2.2. Determination of SOD, POD, APX Activities and Proline Content

The 0.1 mL supernatant extract, same as the method of O_2_^−^, was mixed with 1.5 mL PBS (50 Mm, pH 7.8), 0.3 mL methionine (130 mM), 0.3 mL NBT (750 µM), 0.3 mL EDTA-Na_2_ (100 µM), 0.3 mL riboflavin (20 µM), and 0.5 mL distilled water. The mixture was placed in an incubator at 30 °C under 4000 Lux light intensity for 15 min [37]. In parallel, another mixture containing 0.1 mL distilled water instead of the supernatant was placed separately in the dark and light designed as a control. The OD was measured at 560 nm using the luminometer, as described above. The amount of enzyme required for inhibiting 50% reduction in NBT photochemical within 1 min per gram of FW was determined using the SOD activity (U/g FW/min), which is calculated using Formula (3).

SOD activity (U/g FW/min) = (*OD*_0_ − *OD_s_*) × *V_s_*/(*OD*_0_ × 0.5 × *V_r_* × *FW*) (3)

where *OD*_0_ and *OD_s_* are the absorbances of control under light and measurement for treatment, respectively; and the others are the same as described in the O_2_^−^ content.

The POD activity was measured using the procedure of guaiacol reduction [38]. The 50 μL enzyme solution that was extracted the same as the method of O_2_^−^ and mixed with 3 mL reaction mixture containing 50 mL PBS (0.2 mM, pH 6.0), 19 μL 30.0% H_2_O_2_, and 28 µL guaiacol was measured quickly at 470 nm every 30 s for 2 min by a spectrophotometer (SP-1920, Shanghai Spectral Instrument Co., Ltd., Shanghai, China). The OD_470_ was expressed as a U/g FW/min (Formula (4)).

POD activity (U/g FW) = (Δ*OD* × *V_s_*)/(*FW* × *V_t_* × 0.01 × *t*) (4)

where Δ*OD* is the change in absorbance in 2 min (*t*); and the others are the same as described in the O_2_^−^ content.

The APX activity was determined using the slightly modified procedure of ascorbic acid oxidation [39]. Approximately 2.6 mL PBS (50 mM, pH 7.0, containing 0.1 mM EDTA-Na_2_), 0.15 mL ascorbic acid (5 mM), and 0.15 mL H_2_O_2_ (20 mM), were successively added into 0.1 mL of the supernatant (same as O_2_^−^). Then, the OD_290_ was measured every 30 s for 2 min using a spectrophotometer (as described above). The reduction in the OD_290_ was expressed as a U/g FW/min (Formula (5)).

APX activity (U/g FW) = (Δ*OD* × *V_s_*)/(*FW* × *V_t_* × 0.01 × *t*) (5)

where Δ*OD* is the change in absorbance in 2 min (*t*); and the others are the same as described in the O_2_^−^ content.

To extract proline, 0.2 g ground fresh leaf was homogenized in 2.0 mL sulfosalicylic acid and then boiled for 10 min [40], whereafter was centrifuged at 10,000× *g* for 10 min at 4 °C. Then, 0.2 mL supernatant was added with 0.2 mL glacial acetic acid, 0.4 mL acid ninhydrin, and 0.2 mL sulfosalicylic acid and then boiled for 30 min. After cooling, the mixture was mixed with 0.4 mL toluene and then centrifuged at 900× *g* for 5 min at 4 °C. The OD was measured at 520 nm using as described above luminometer.

Proline content (μg/g FW) = (*X* × *V_s_*)/(*FW* × *V_t_*) (6)

Annotation is the same as described in the O_2_^−^ content.

#### 2.2.3. Assessment of Morphological Characteristics and Crop Biomass

Three pots containing nine plants with uniform growth were selected for measuring LA/plant and plant height by a straightedge and stem diameter by a digital vernier caliper (DL3944, Ningbo Deli Tools Co., Ltd., Zhejiang, China). Stem and leaf tissues were separately dried at 108 °C for 30 min and then roasted at 75 °C until reaching a constant weight, and the dry matter for stem and leaf was determined. The LA was determined from Formula (7).

(7) $$\mathrm{LA}=0.75\;\times \;\sum_{i=1}^{m}\sum_{j=1}^{n}\;(L_{\mathrm{ij}}\;\times \;W_{\mathrm{ij}})/m$$

where 0.75 is the empirical correction coefficient for LA of maize; *m* is plant number per replication (three plants); *n* is the total number of leaves on the *i*th plant; and *L_ij_* and *W_ij_* are the maximum leaf length and width of *j*th leaf in the *i*th plant_,_ respectively.

#### 2.2.4. Measurement of Leaf Relative Water Content

Top fully developed fresh leaves in three plants per replication were weighed using a 1/1000 balance recorded as FW. Then, the leaves were immersed in water for 8 h. After soaking in the surface water, the leaves were weighed to determine their turgid weight. Subsequently, the turgid leaves were placed in an oven at 108 °C for 30 min before drying at 75 °C to a constant weight to record the dry weight. The leaf relative water content (RWC) was calculated from Formula (8) [41].

RWC (%) = (*FW* − *Dry weight*)/(*Turgid weight* − *Dry weight*) × 100 (8)

#### 2.2.5. Tolerance Analysis

The comprehensive evaluation (D) value and tolerance coefficient were adopted to predict the tolerance of varieties under WS. The SOD, POD, APX, proline, LA, stem diameter, plant height, and dry matter were involved in the calculation of the D value and tolerance coefficient using the following formulas [42].

(9) $$u(X_{\mathrm{ij}})=(X_{\mathrm{ij}}-{\;X}_{\min})/\left(X_{\max}-{\;X}_{\min}\right)$$

(10) $$V_{j}=\sqrt{\sum_{i=1}^{n}{\left(X_{\mathrm{ij}}-{\bar{X}}_{j}\right)}^{2}}/{\bar{X}}_{j}$$

(11) $$W_{j}={\;V}_{j}/\sum_{j=1}^{n}V_{j}$$

(12) $$D=\sum_{j=1}^{n}[u(X_{j})\;\times {\;W}_{j}]$$

(13) $$\mathrm{Tolerance}\;\mathrm{coefficient}=\sum_{j=1}^{n}({\mathrm{Stress}}_{j}/{\mathrm{CK}}_{j})/n$$

where the value of *i*th variety in the *j*th indicator was *X_ij_*; the membership function value, maximum value, minimum value, standard deviation coefficient, and average value for the *j*th indicator of two varieties were *μ*(*X_ij_*), *X_max_*, *X_min_*, *V_j_* and ${\bar{X}}_{j}$, respectively, and the weight of the *j*th indicator in all indicators was *W_j_*.

### 2.3. Statistical Analysis

The software of SAS 9.2 (SAS Institute Inc., Cary, NC, USA) was used to carry out the one-way analysis of variance (ANOVA) for GB concentration, three-way ANOVA for variety, and the WS and GB concentration to determine the interaction between each factor, and the Pearson’s correlation analysis among all parameters. Means with *n* = 3 biological replicates for all indicators were compared using the least significant difference (LSD) at *p* ≤ 0.05. The stepwise regression analysis among SOD, POD, APX, proline, LA, plant height, stem diameter, and stem dry matter with leaf dry matter was performed by the software of SPSS Statistics v. 21 (IBM Inc., Armonk, NY, USA). Moreover, figures were generated using OriginPro 2021 (OriginLab Inc., Northampton, MA, USA).

## 3. Results

### 3.1. Glycinebetaine Reduced the Accumulation of Reactive Oxygen Species under Water Stress

**Figure 2:**
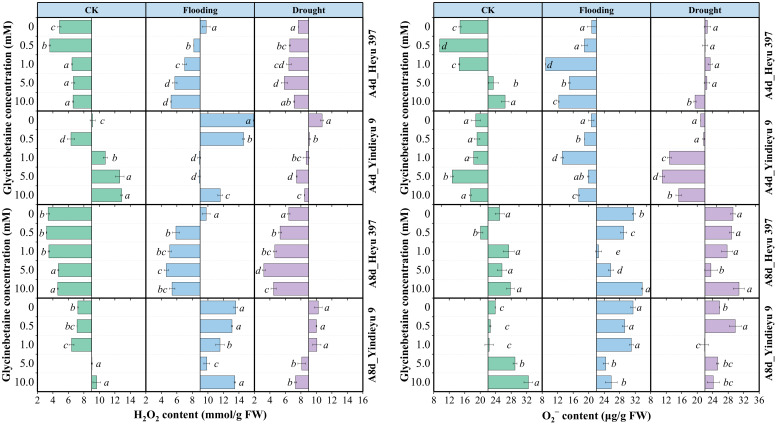
The effects of glycinebetaine on the accumulations of hydrogen peroxide (H_2_O_2_) and superoxide anion (O_2_^−^) after 4 d (A4d) and 8 d (A8d) of water stress. CK represents maize planted in normal field capacity; bars represent standard error (*n* = 3, biological replicates); different letters in a water treatment indicate the least significant differences as *p* value ≤ 0.05.

Without the influence of GB, prolonged WS and growth stage resulted in a marked increase in O_2_^−^ content but a decrease in H_2_O_2_ content (Figure 2). After 8 d, the CK without GB reduced the H_2_O_2_ content by 25.52%, with an increased O_2_^−^ content by 47.99%, compared with those after 4 d. Yindieyu 9 accumulated more H_2_O_2_ content and less O_2_^−^ content under WS, but showed less increment ratio in H_2_O_2_ (65.00%) compared with Heyu 397 (140.83%) without GB. In addition, the H_2_O_2_ and O_2_^−^ contents under flooding stress without GB were ~1.42 fold and ~1.15 fold higher when compared with those under drought after 8 d of stress. The accumulation of ROS was significantly affected not only by WS but also by GB and the interaction between them (*p* < 0.01). The generation of ROS in all treatments was first attenuated and then rose with an increase in the concentration of GB. Although ROS in CK under a high dosage of GB was increased, CK still exhibited lower levels of ROS compared with WS, in which the effective concentration of GB for eliminating ROS in CK was GB1. Meanwhile, the ROS under WS was significantly reduced by GB (*p* < 0.05), but the ROS under flooding stress was still higher than that under drought. The optimum dosage of GB for ROS after the 8 d stress was detected at GB3. Meanwhile, more GB (~5.0 to 10.0 mM), especially for Heyu 397, was needed under the progressive WS to decrease the O_2_^−^ content. After 8 d, under CK, flooding, and drought, GB promoted ROS content by 9.65%, −29.34%, and −18.36% in the variety Heyu 397, respectively, and, correspondingly, by 11.33%, −11.89%, and −7.83% in the variety Yindieyu 9, compared with GB0. As a result, GB was more beneficial in attenuating the accumulation of ROS under flooding stress, especially for Heyu 397.

### 3.2. Glycinebetaine Was More Beneficial to Improving SOD Activity under Flooding Stress

**Figure 3:**
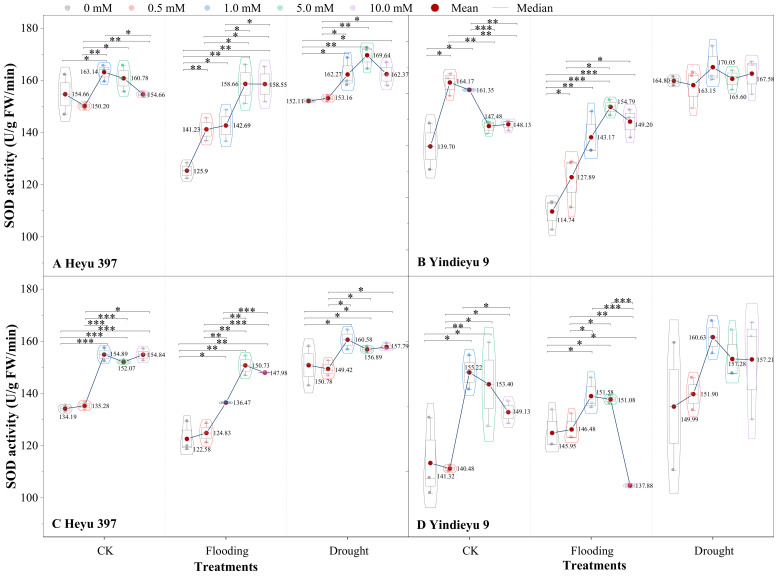
The effect of glycinebetaine on superoxide dismutase (SOD) activity after 4 d (**A**,**B**) and 8 d (**C**,**D**) of water stress. Data with standard error bars (*n* = 3, biological replicates) are presented; CK represents maize planted in normal field capacity; the 0, 0.5, 1.0, 5.0, and 10.0 mM are the different concentrations of glycinebetaine; *, **, *** mean significant *p* value ≤ 0.05, ≤0.01, ≤0.001, respectively, if no symbol is presented between treatments, meaning *p* value > 0.05.

The interaction of either WS or the variety with GB significantly affected SOD activity (Figure 3, *p* < 0.01). Compared with that after 4 d, under CK, flooding, and drought, the SOD activity without GB after 8 d for the Heyu 397 was decreased by 13.24%, 2.24%, and 0.87%, respectively, and in Yindieyu 9 was increased by 1.16%, 27.21%, and −8.99%, respectively. When maize seedlings were exposed to WS, the SOD activity exhibited a significant decrease under flooding, while an increase under drought, compared with CK. The GB played a significant role in improving the SOD activity in all treatments (*p* < 0.001). After 8 d, under the effect of GB, drought still had a significantly higher SOD activity than flooding and CK (*p* < 0.05), while with flooding there existed a lower SOD activity than CK. The SOD activity first increased and then descended in response to the increase in concentration of GB. The greatest increment in SOD activity was discovered at GB3 under flooding and drought in Heyu 397, at GB3 (after 4 d) and GB2 (after 8 d) in Yindieyu 9, and at GB2 in CK in both varieties. After 8 d, Yindieyu 9 exhibited a higher SOD activity than Heyu 397 under WS and CK, particularly under flooding, but the high SOD activity in Yindieyu 9 under drought was unaffected by GB. After 8 d, compared with GB0, under CK, flooding, and drought, SOD activity under the effect of GB increased in Heyu 397 by 11.24%, 14.21%, and 3.57%, respectively, and, correspondingly, in Yindieyu 9 by 5.38%, 0.55%, and 4.51%, respectively. As a consequence, GB was more beneficial in improving the SOD activity of Heyu 397 under flooding.

### 3.3. Glycinebetaine Enhanced the POD Activity under Water Stress

**Figure 4:**
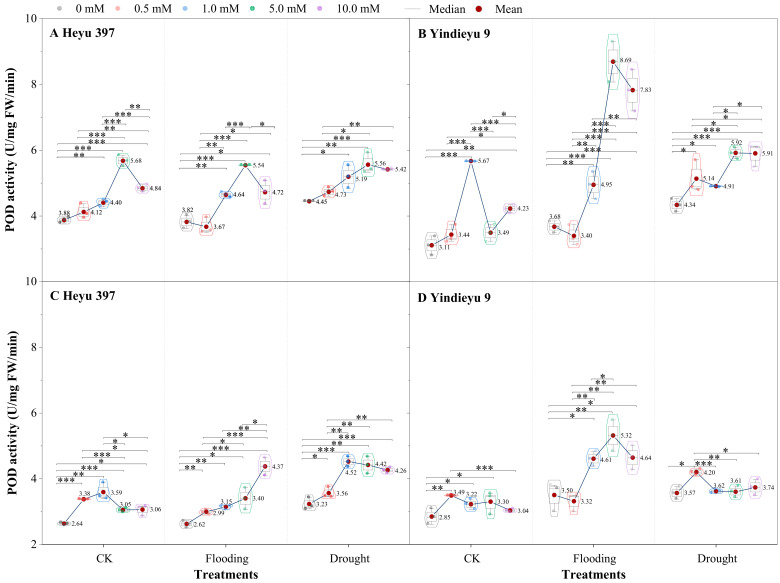
The effect of glycinebetaine on guaiacol peroxidase (POD) activity after 4 d (**A**,**B**) and 8 d (**C**,**D**) of water stress. Data with standard error bars (*n* = 3, biological replicates) are presented; CK represents maize planted in normal field capacity; the 0, 0.5, 1.0, 5.0, and 10.0 mM are the different concentrations of glycinebetaine; *, **, *** mean significant *p* value ≤ 0.05, ≤0.01, ≤0.001, respectively, if no symbol is presented between treatments, meaning *p* value > 0.05.

From 4 d to 8 d and in the absence of GB, the POD activities under CK, drought, and flooding declined in Heyu 397 by 31.97%, 31.27%, and 27.32%, respectively, and, correspondingly, in Yindieyu 9 by 8.50%, 4.69%, 17.78%, respectively (Figure 4). After 8 d, compared with CK in the absence of GB, WS greatly improved POD activity, especially drought stress, which increased the POD activity by 22.62% in Heyu 397 and 25.30% in Yindieyu 9. After 8 d, Heyu 397 showed significantly higher POD activity under drought than under flooding and CK (*p* < 0.05), while Yindieyu 9 had no significant difference in the POD activity between drought and flooding (*p* > 0.01). Moreover, Yindieyu 9 had higher POD activity than Heyu 397 (*p* < 0.05). Foliar application of GB significantly increased POD activity no matter what the water conditions (*p* < 0.01), where the rank for POD activity from high to low was drought, flooding, and CK in Heyu 397, and flooding, drought, and CK in Yindieyu 9 after 8 d. The POD activity was first significantly improved and then declined with the increase in the concentration of GB (*p* < 0.01), revealing that a high concentration of GB (GB4) inhibited the infinite increase in POD activity, but still improved the POD activity compared with CK. After 4 d, the highest POD activity was observed at GB3 in all treatments, except for GB2 in CK of Yindieyu 9. Nevertheless, after 8 d, the highest POD activity was observed at GB2 under CK and drought, at GB4 under flooding in Heyu 397, and, correspondingly, at GB1 under CK and drought, and at GB3 under flooding in Yindieyu 9. After 8 d, compared with GB0, the POD activities under CK, drought, and flooding were increased by the foliar GB by 24.02%, 32.56%, 29.64% in Heyu 397, respectively, and by 14.62%, 27.58%, and 6.25% in Yindieyu 9, respectively. Results indicated that GB contributed more POD activity towards flooding, as well as Heyu 397 than Yindieyu 9.

### 3.4. Glycinebetaine Improved the APX Activity under Water Stress

**Figure 5:**
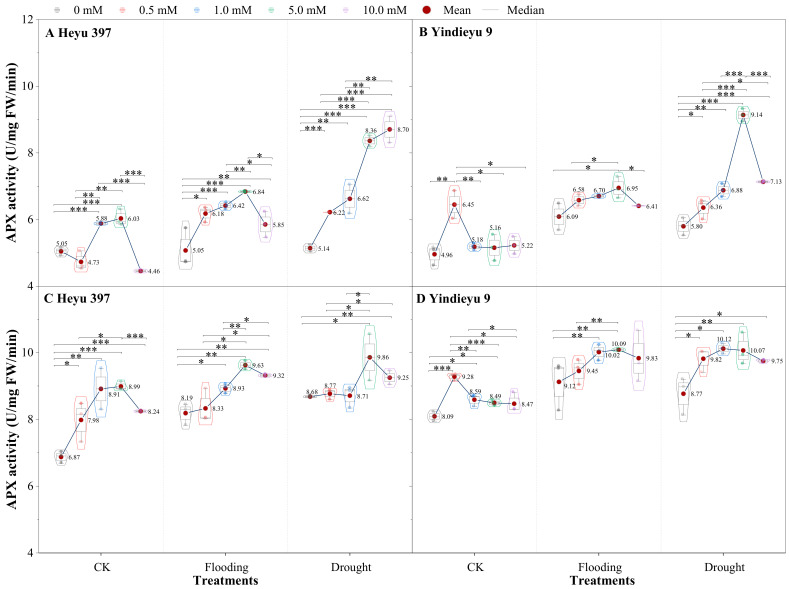
The effect of glycinebetaine on ascorbate peroxidase (APX) activity after 4 d (**A**,**B**) and 8 d (**C**,**D**) of water stress. Data with standard error bars (*n* = 3, biological replicates) are presented; CK is maize planted in normal field capacity; 0, 0.5, 1.0, 5.0, and 10.0 mM are the different concentrations of glycinebetaine; *, **, *** mean significant *p* value ≤ 0.05, ≤0.01, ≤0.001, respectively, if no symbol is presented between treatments, meaning *p* value > 0.05. Data.

Prolonged WS and growth stage induced an obvious rise in APX activity (Figure 5). Compared with after 4 d, APX activities after 8 d stress under CK, drought, and flooding were increased by 36.21%, 45.19%, and 57.74% in Heyu 397, respectively, and 63.20%, 56.97%, and 51.29% in Yindieyu 9, respectively. Yindieyu 9 accumulated higher APX activity under flooding relative to drought in the absence of GB after 8 d. In contrast to Yindieyu 9, Heyu 397 displayed lower APX activity under WS and CK and maintained higher APX activity under drought than under flooding. Nevertheless, there was no significant difference between drought and flooding treatments in APX for plants (*p* > 0.05). Water stress and GB significantly affected APX activity (*p* < 0.01), but the interaction between the two factors had no obvious effect on APX activity (*p* > 0.05). Throughout the whole period of treatment for Heyu 397, the highest APX activity was found at GB3, which was similar to that at GB2 under CK, and at GB3 under WS; they were all significantly higher than at other concentrations of GB (*p* < 0.05). Meanwhile, in Yindieyu 9, the highest APX activity was detected at GB1 under CK and at GB3 under WS, but there was no significant difference between treatments with GB after 8 d (*p* > 0.05). It was also revealed that Yindieyu 9, under the effect of GB, still had significantly higher APX activity than Heyu 397 after 8 d (*p* < 0.01). Although a low dosage of GB was suitable for CK, high GB still improved the APX activity compared with the treatments without GB, and so conducted under WS. After 8 d, compared with GB0, GB was more beneficial for improving APX activity under flooding in Heyu 397, with about a 10.53% increase and under drought in Yindieyu 9 by 13.33%.

### 3.5. Lower Dosage of Glycinebetaine Increased the Proline Content under Water Stress

Proline content steadily declined with the progression of WS and growth. Proline content of Yindieyu 9 showed a higher reduction from 4 d to 8 d, compared with Heyu 397. Furthermore, proline content without GB after 8 d of stress significantly decreased under flooding and significantly increased under drought (∼7-fold as compared with CK) (Table 1, *p* < 0.01). Yindieyu 9 accumulated less proline content than Heyu 397 in the absence of GB. Although GB significantly promoted the proline content under WS (*p* < 0.01), high concentrations of GB had adverse effects on maize leaves under CK because the proline content declined under CK after 8 d. With prolonged WS, maize’s growth needed more and more GB to produce more proline. There was no significant difference in proline content induced by GB between flooding and CK (*p* > 0.05), which were all lower than drought. Proline content promoted by GB was significantly higher in Heyu 397 in comparison with Yindieyu 9. The proline content was enhanced most by GB2 under CK, GB1 under flooding, and GB3 under drought in Heyu 397, correspondingly, by GB2, GB2, and GB1 in Yindieyu 9 after 4 d of stress. After 8 d, the highest increment in proline content, under WS from the effect of GB, was detected at GB3, of which, the average increment was 11.74% under flooding and 136.95% under drought in Heyu 397, and 11.93% under flooding and 99.68% under drought in Yindieyu 9. Consequently, GB contributed more proline content towards Heyu 397, especially for treatments under drought.

### 3.6. Glycinebetaine Promoted the Growth of Maize Seedlings under Water Stress

**Figure 6:**
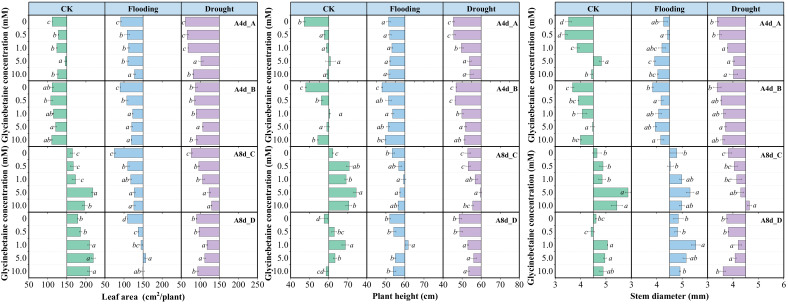
The effect of glycinebetaine on maize morphology after 4 d (A4d) and 8 d (A8d) of water stress. CK represents maize planted in normal field capacity; the letters A and C are the variety Heyu 397, and B and D represent the variety Yindieyu 9; bars represent standard error (*n* = 3, biological replicates); different letters in a water treatment indicate the least significant differences as *p* value ≤ 0.05.

From 4 d to 8 d, LA and stem diameter in the absence of GB under flooding stress had a higher increment than under drought stress, while plant height showed the exact opposite trend. Although there was no difference in the average increment between Yindieyu 9 and Heyu 7 under drought, a higher average increment occurred under flooding in Yindieyu 9 than in Heyu 397. Meanwhile, after 8 d, maize growth was significantly suppressed when subjected to drought in the absence of GB, resulting in smaller LA, thinner stem diameter, and shorter plant height compared with CK (Figure 6, *p* < 0.05). By contrast, maize under flooding exhibited a thicker stem diameter, shorter plant height, and smaller LA than CK after 8 d. Although the difference in plant height between drought and flooding was not statistically significant, flooding resulted in higher plant height, stem diameter, and LA than drought in the absence of GB after 8 d (*p* > 0.05). Meanwhile, Yindieyu 9 maintained a significantly larger stem diameter and LA compared with Heyu 397 (*p* < 0.05). Foliar application of GB significantly mitigated the adverse effects of WS on maize growth, subsequently promoting crop growth (*p* < 0.05). With the progression of growth, GB took a more effect on LA and plant height and less of an effect on stem diameter. However, the difference in morphological indicators affected by GB in WS and variety was similar to that without GB after 8 d. The morphological indicators were first increased and then reduced with an increase in GB concentration in all treatments. However, under the effect of GB, all morphological indicators were higher than those at GB0. After 8 d, to promote growth to survive from WS, Yindieyu 9 (GB2) required less GB than Heyu 397 (GB3) to improve the stem diameter, while there was a similar and optimum dosage of GB that GB3 required between Yindieyu 9 and Heyu 397 to promote LA and plant height. Under CK, the optimum concentration for maize growth was GB3 after four times foliar application of GB, and other concentrations still maintained higher morphological indicators in comparison with GB0. The GB had the greatest effect on LA, followed by plant height, and had the lowest effect on stem diameter. Whereas, GB was more beneficial to increasing LA in Heyu 397 under flooding.

### 3.7. Glycinebetaine Increased the Biomass Accumulation of Maize Seedlings under Water Stress

**Figure 7:**
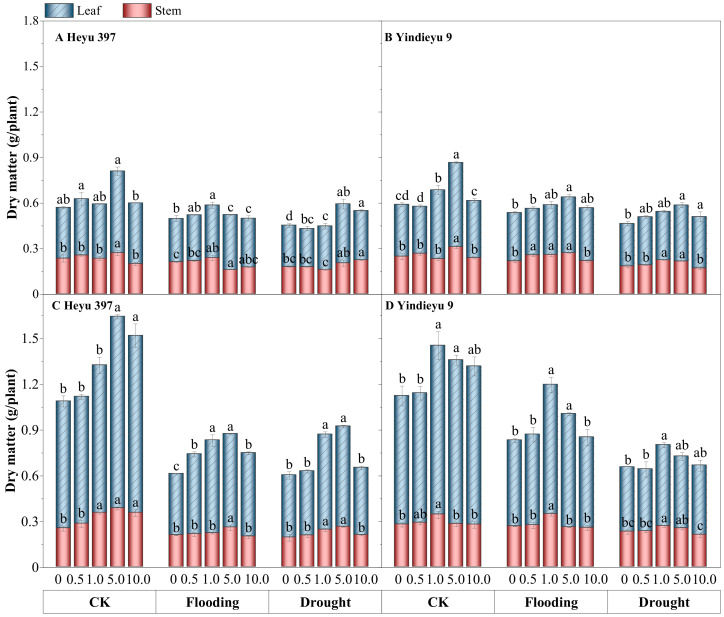
The effect of glycinebetaine on stem and leaf biomass accumulation after 4 d (**A**,**B**) and 8 d (**C**,**D**) of water stress. CK represents maize planted in normal field capacity; the 0, 0.5, 1.0, 5.0, and 10.0 mM are the different concentrations of glycinebetaine; vertical bars represent standard error (*n* = 3, biological replicates); different letters in a water treatment indicate the least significant differences as *p* value ≤ 0.05.

Water stress significantly slowed down the overall growth of maize seedlings, particularly in terms of leaf dry matter, compared with CK (Figure 7, *p* < 0.05). From 4 d to 8 d, under CK and with drought and flooding stresses in the absence of GB, the total dry matter had grown by 78.18%, 19.81%, and 28.76% in Heyu 397, respectively, and 79.46%, 49.93%, 38.55% in Yindieyu 9, respectively, in which leaf dry matter was much more severely reduced by WS, especially under flooding stress. Additionally, stem dry matter under flooding in the absence of GB was reduced by 17.65% in Heyu 397 and 4.17% in Yindieyu 9 after 8 d, although not statistically significant between flooding and CK (*p* > 0.05). Drought also caused a significant reduction in leaf dry matter compared with CK (*p* < 0.05), which was also lower than flooding. Meanwhile, higher stem biomass was also observed under flooding rather than drought, with no significant difference (*p* > 0.05). Similarly, Heyu 397 showed significantly lower total biomass than Yindieyu 9 under flooding (*p* < 0.05), and no statistically significant difference compared with Yindieyu 9 under drought. The adverse effects of WS on the biomass accumulation of maize seedlings were significantly and steadily reduced with the increasing in GB concentration (*p* < 0.05). The most significant increment in biomass accumulation was observed at GB3 after 4 d, while was at GB3 with Heyu 397 and GB2 with Yindieyu 9 after 8 d. Although there was no adverse effect of GB on biomass accumulation of maize seedlings in all treatments, the higher dosage of GB had an inhibitory effect on biomass accumulation. Additionally, GB had contributed more biomass accumulation towards Heyu 397 than Yindieyu 9 especially in terms of leaf dry matter, which showed an increment of 21.95% under drought and 42.71% under flooding in Heyu 397. It also led to a similar dry matter between drought and flooding in Heyu 397, which was significantly lower than Yindieyu 9.

### 3.8. Glycinebetaine Improved Leaf Relative Water Content under Drought Stress

Compared with CK, RWC was not significantly altered under flooding (*p* > 0.05) but was significantly reduced during drought (Table 2, *p* < 0.01). Under drought without GB, the RWC of Heyu 397, with a decrement of 28.67%, was significantly lower than that of Yindieyu 9 (*p* < 0.05). Foliar application of GB significantly increased RWC under drought by 22.90% in Heyu 397 and 13.23% in Yindieyu 9 (*p* < 0.05) but had no apparent impact on RWC under flooding and CK (*p* > 0.05). The most suitable concentration for plants grown under drought was GB3. Whereas, the higher dosage of GB had an inhibitory but non-toxic effect on the RWC of maize.

### 3.9. Analysis for Correlation, Stepwise Regression, and Tolerance

**Figure 8:**
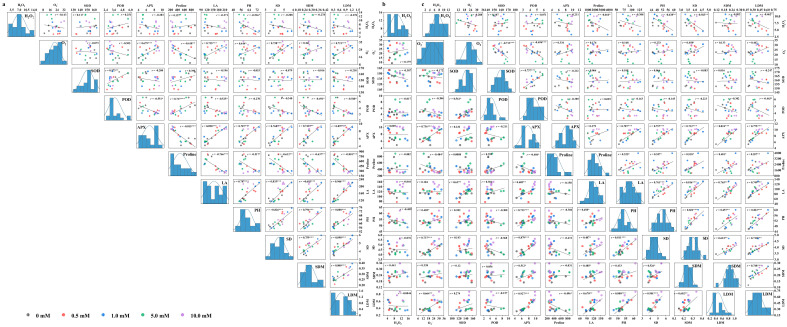
Correlation analyses of the inspected parameters of maize under CK (**a**), flooding (**b**), and drought (**c**) stresses in the influence of glycinebetaine. CK represents maize planted in normal field capacity; Correlation coefficient *r* with the least significant difference according to *p* value ≤ 0.05 (*, **, *** mean significant *p* value ≤ 0.05, ≤0.01, ≤0.001) is shown. H_2_O_2_, O_2_^−^, SOD, POD, APX, LA, PH, SD, SDM, and LDM represent hydrogen peroxide, superoxide anion, superoxide dismutase, guaiacol peroxidase, ascorbate peroxidase, leaf area, plant height, stem diameter, stem dry matter, and leaf dry matter, respectively.

The correlation analysis revealed that the correlations among most of the indicators under WS were similar to those in CK (Figure 8). However, SOD activity under flooding and proline content under drought showed the opposite correlation with some other indicators compared with CK, whereby either SOD activity under flooding or proline content under drought was positively correlated with morphological parameters and biomass. Additionally, POD activity significantly and positively correlated with SOD activity (*p* < 0.05). Furthermore, in all treatments, APX activity showed a significant positive correlation with morphological parameters and biomass (*p* < 0.05). Leaf area, plant height, stem diameter, and dry matter were significantly and positively correlated with each other (*p* < 0.05). It was APX activity under flooding, POD under drought, and plant height under both stresses that, screened out by the stepwise regression analysis, were the critical and representative indicators for leaf dry matter, which could determine a relatively high mean forecast accuracy of more than 80% (Table 3). Finally, the D value, as an important indicator to evaluate water tolerance, was detected to be the highest in CK, followed by drought and flooding (Table 4). The tolerance coefficient was more than 1.0 under drought, while lower than 1.0 under flooding. Yindieyu 9 had a higher tolerance to flooding and drought than Heyu 397, and its tolerance to flooding was ~3.17 fold as compared with Heyu 397 based on the D value.

## 4. Discussion

Global climate change has resulted in an accelerating number of intense alternating drought and flooding incidents, which are extremely expensive in terms of crop production loss [43,44]. It is also a major factor limiting maize production in the subtropical region of Guangxi, China. Few previous studies have compared the differences and similarities in responses of different maize genotypes under flooding and drought stresses. It is urgent to understand the adaptive mechanism and search for effective techniques to avoid or mitigate the damaging effect of both drought and flooding on maize growth in the area. A significant amount of ROS buildup occurred when maize suffered from WS, similar to previous studies [12,45,46]; however, regardless of WS or not, plant development duration increased O_2_^−^ and decreased H_2_O_2_ accumulation, which was different from previous studies that maize had accumulated a higher ROS at V3 than V5 [12,16] or gradually improved with growth stage and stress duration [18,47]. The distinctly different responses of maize may result from a self-regulation compensation mechanism of different genotypes to enable plants to tolerate the damage from WS or the environment [48]. The self-regulation of maize in the study was reflected in the increase in SOD and POD activities and the decrease in APX activity from 4 to 8 d WS or growth, as was consistent with previous reports [49,50]. A low SOD activity led the plants to convert less O_2_^−^ to H_2_O_2_. Meanwhile, although a high POD activity was detected, high APX has been identified as a key antioxidant enzyme to contribute the most tolerance to WS [3,45], as well as stress duration, which could still convert more H_2_O_2_ to oxygen. Eventually, more O_2_^−^ and less H_2_O_2_, accompanied by a higher increment of O_2_^−^ than the reduction of H_2_O_2_, were accumulated with the progression of WS in the study.

Drought and flooding are two of the key environmental factors that cause varying degrees of oxidative damage to maize growth. Maize exposed to drought in the absence of GB had a more significant increment in the antioxidative enzyme activities of SOD, POD, and APX and proline content. By contrast, flooding just increased POD and APX activities, which accelerated the conversion of H_2_O_2_, while decreasing SOD activity and proline content compared with CK. Azahar et al. [13] also reported that maize shows a significant decrement in the activities of SOD and other antioxidant enzymes involved in the ascorbate-glutathione (AsA-GSH) cycle, causing deregulation of the ROS scavenging system when maize suffered from flooding. Consequently, flooding deregulated ROS scavenging machinery accompanied by an accumulation of ROS associated with lower SOD and POD activities and proline content and ultimately reduced the tolerance of maize, showing a lower D value compared with drought. In addition, it was also further confirmed in this study that it was APX under flooding and POD under drought, screened out by a stepwise regression analysis, that played the key role in maize to combat the WS damages, similarly proposed by prior studies [3,45,46]. Moreover, APX activity was significantly and positively correlated with morphological parameters and biomass in all treatments, also revealing its importance in resisting WS. The aboveground plant parts exhibit growth inhibition under WS and gradually undergo a series of morphological structure changes to adapt to WS [51]. In this study, it was found that four days of short flooding caused less damage to plant growth than drought, as indicated by a higher LA and stem diameter during flooding compared with those under drought. In particular, the highest stem diameter occurred in flooding in this study, which has been confirmed to be one of the important elements that prevent plants from lodging to adoptive growth under flooding stress [16,52]. Although flooding had higher LA than drought, leaf dry matter showed no significant difference between flooding and drought due to the higher RWC under flooding. Even though maize leaves kept lower oxidation tolerance to flooding, short-term flooding of 4 d gave rise to a lighter effect on the leaf growth than drought. The flooding duration is probably short enough to avoid noticeable damage to root and electron transfer; short-term flooding can promote the photosynthetic rate of leaves and the uptake of nutrients by plant roots [53,54]. Certainly, on the other hand, long-term flooding of 8 d generated serious damage to growth, decreasing the increment of morphological characteristics and biomass compared with normal growth. The lowest RWC was observed under drought in this study, which has been previously proved to restrict stomatal conductance to inhibit photosynthetic rate [51], and thus suppress growth leading to a decrease in morphology characteristic and biomass. Overall, maize growing under flooding mainly relies on POD and APX activities to scavenge ROS and maintain a stronger stem, showing a weaker tolerance than under drought, while under drought mainly depends on antioxidative enzyme activities together with proline to eliminate ROS and resist stress for survival. However, maize adoptive growth depends not only on SOD, POD, and APX activities and proline content but also on other enzymes in the AsA-GSH cycle and osmotic adjustment substances [12,13], as it is a comprehensive and complex regulatory mechanism for plants to combat WS.

However, the response of maize to drought and flooding had been slightly changed with genotypes. Yindieyu 9 before Heyu 397 had been identified to be more tolerant to both drought and flooding, but both varieties demonstrated a greater capacity for drought than flooding based on the D values (a comprehensive performance for all indicators) and tolerance coefficients. The tolerant variety demonstrated a greater antioxidant capacity and higher morphological characteristics (except plant height) and dry matter than the sensitive variety. Previous studies had reported that a tolerant cultivar to drought is also tolerant to waterlogging [3,55], which was also verified in Yindieyu 9. With the lengthening of stage or stress, a distinct response for variety was revealed; Yindieyu 9 had a higher average increment of SOD, POD, and APX activities, morphological characteristics, and dry matter under WS, especially for flooding in comparison with Heyu 397. Heyu 397 possessed higher antioxidant activities and dry matter under drought relative to flooding, even though it was a sensitive genotype to drought and flooding. Therefore, the water-tolerant variety not only had a high ability to suffer from serious stress but also long-term stress.

Limit research has provided us with a limited understanding of the differential roles GB plays in the defense against drought and flooding stresses, even though GB has been applied in many plants under abiotic stress [20,21,25]. Therefore, this study was carried out to focus on the role of GB in various susceptible genotypes under either drought or flooding stress. This study revealed that, whether under WS or not, foliar application of GB significantly increased SOD, POD, and APX activities, thereby enhancing the antioxidant capacity of maize in all treatments. GB contributed the most to the POD activity, with the highest recrement among the antioxidants for maize, which enhanced the crucial role in the H_2_O_2_ scavenging system. In addition, it is revealed that exogenous GB significantly increased proline content, which mainly plays an important role in osmoregulation in plants under drought stress [19]. Proline was positively correlated with morphological parameters and biomass under drought, while negatively correlated under CK and flooding conditions. The possible reason may be that the lower proline content under flooding and CK has less effect on the growth of maize, but GB can improve proline content and therefore reinforce the role of proline in flooding. Previous studies have also reported a similar increase induced by GB in antioxidant activity and osmolyte accumulation [11,26]. The GB collaborated with the antioxidant activity and osmolyte accumulation in the leaves alleviating the oxidative damage caused by excess accumulation of ROS under WS. In the current study, the average contents of H_2_O_2_ and O_2_^−^ were severely decreased by 25.82% and 7.81% under the function of foliar application of GB under WS. It is also indicated that exogenous GB was more beneficial to scavenging excess H_2_O_2_ under WS, which was associated with the essential role of POD activity induced by GB, as suggested above. In addition, exogenous GB significantly improved leaf RWC under drought which was consistent with previous results [23] but did not affect RWC under flooding as flooding induced no significant differences in RWC relative to the CK. High levels of antioxidant activity, osmolyte accumulation, and RWC (drought) and a low level of ROS accumulation under WS-induced foliar-GB promoted maize growth. Thereby, the LA, plant height, stem diameter, and dry matter for leaves and stems under WS or not had been improved under the effect of GB, mediating with antioxidants and proline. It was also revealed that GB contributed the largest effect to LA, with the highest increment relative to other morphological characteristics, followed by plant height, resulting in the largest effect on dry matter for leaf rather than stem. The plant height was also screened out as an efficient indicator for leaf dry matter under WS, as shown in other reports [3,56].

The effects of exogenous GB on plants greatly varied according to crop cultivar, stress duration, stress type, and GB concentration. Despite previous studies that reported different or the same roles of GB taking in tolerant cultivars and sensitive cultivars [23,30,31], the role is mainly researched based on the same stress. The reports regarding the different effects of foliar-applied GB on different tolerant cultivars to drought and flooding stresses have been limited, so far. When the exogenous GB was sprayed to leaves after 8 d, maize still maintained higher oxidation tolerance to drought, associated with higher SOD, POD, and APX activities, proline content, and ROS accumulation, accompanied by no statistical difference in morphological characteristics and dry matter compared with flooding, revealing that although GB could not change the trend of each index, it could shorten the difference induced by drought and flooding. Nevertheless, the deleterious effect of flooding on maize, rather than drought and CK, was mitigated more by foliar GB. Heyu 397, which is more sensitive to WS, especially to flooding than Yindieyu 9, was more enhanced by GB, which was different from the previous study [23]. Regardless of the variety, under WS and CK stresses, antioxidant activities, proline (except CK), morphological characteristics, RWC, and biomass accumulation were all first increased and then reduced with the increase in the concentration of exogenous GB. Nevertheless, an excessive dosage of exogenous GB, which reached 10 mM in this study, exhibited inhibitory but non-toxic effects on maize seedlings. A high concentration of GB under WS or not still promoted the physiological and biochemical characteristics and growth of maize when compared with the treatment in CK without GB. In addition, a lower dosage of exogenous GB was generally needed for the water-tolerant variety Yindieyu 9 under flooding accompanied by the lengthening of stress duration relative to the sensitive variety Heyu 397 or vice versa. The most effective and efficient dosage of GB was determined to be 5.0 mM under WS, which also greatly improved the growth of maize grown in CK. GB biosynthesis is closely linked with ethylene biosynthesis by the common mediator molecule of choline, but GB production might occur at the expense of ethylene synthesis due to less transfer from S-adenosyl methionine to choline [29]. In addition, choline and ethylene play important roles in plant tolerance to stress [57,58]. Thus, the important metabolic pathway involving the relationship among GB, hormones, and defense system needs to be studied further.

## 5. Conclusions

Maize exhibited greater drought tolerance owing to higher antioxidant activities and proline content, and lower reactive oxygen species in comparison with flooding. It might be the decrease in the superoxide dismutase activity and proline content that led to a larger accumulation of reactive oxygen species under flooding. The different responses to drought and flooding would be slightly changed by genotypes and stress duration. Specifically, guaiacol peroxidase under drought and ascorbate peroxidase under flooding were significant variables for the accumulation of leaf dry matter. Foliar application of GB played an important role in improving tolerance and promoting growth in all treatments, where it contributed the most to POD and leaf growth to eliminate more hydrogen peroxide than superoxide anion. Furthermore, GB had a greater impact on flooding with the water-sensitive variety Heyu 397 compared to the water-tolerant variety Yindieyu 9 under drought, which could shorten the difference induced by drought and flooding. More and more GB was required in Heyu 397 to protect or survive from the damages of water stress, while less GB was needed for maize planted in well-watered conditions with a growth stage. In general, irrespective of the maize variety, 5.0 mM emerged as the most effective and efficient dosage of GB under stress and also promoted maize growth in well-watered conditions. Overall, this study enhances the understanding of the contrasting defense regulation for alleviating oxidative damage in response to drought vs. flooding stresses and highlights the foliar application of GB as a valuable and effective strategy for alleviating water stress-induced damage to the physiochemical attributes and growth of maize.

## Figures and Tables

**Table 1:** The effect of exogenous glycinebetaine (GB) on the proline content (μg/g FW) of maize leaves under water stress.

Water Treatments	GB (mM)	After 4 d		After 8 d	
		Heyu 397	Yindieyu 9	Heyu 397	Yindieyu 9
CK	0	514.03 ± 53.28 ^d^	516.97 ± 32.46 ^b^	433.06 ± 48.68 ^a^	351.74 ± 59.51 ^a^
	0.5	773.46 ± 13.20 ^bc^	484.00 ± 29.75 ^b^	312.15 ± 19.12 ^bc^	264.64 ± 39.26 ^b^
	1.0	914.96 ± 5.76 ^a^	658.77 ± 22.34 ^a^	333.71 ± 10.26 ^b^	257.60 ± 31.10 ^b^
	5.0	731.70 ± 20.56 ^c^	636.56 ± 49.11 ^a^	286.30 ± 2.85 ^c^	263.65 ± 35.82 ^b^
	10.0	805.06 ± 52.51 ^b^	496.00 ± 15.93 ^b^	285.09 ± 11.78 ^c^	218.50 ± 22.50 ^b^
Flooding	0	450.08 ± 51.47 ^b^	483.93 ± 2.55 ^c^	315.87 ± 44.52 ^bc^	271.27 ± 1.07 ^b^
	0.5	765.04 ± 90.03 ^a^	310.40 ± 24.91 ^d^	289.94 ± 14.43 ^c^	290.44 ± 28.11 ^b^
	1.0	408.71 ± 5.24 ^b^	868.75 ± 49.61 ^a^	353.37 ± 17.67 ^b^	285.66 ± 2.24 ^b^
	5.0	359.11 ± 23.79 ^bc^	567.70 ± 44.73 ^b^	424.42 ± 3.39 ^a^	333.84 ± 8.23 ^a^
	10.0	296.97 ± 51.38 ^c^	495.31 ± 49.33 ^c^	344.03 ± 46.18 ^bc^	295.52 ± 15.18 ^b^
Drought	0	1545.92 ± 175.38 ^d^	1472.65 ± 13.69 ^c^	1128.34 ± 42.66 ^d^	1109.00 ± 28.02 ^e^
	0.5	1661.98 ± 37.12 ^cd^	2875.10 ± 65.66 ^a^	2477.36 ± 225.42 ^b^	1963.93 ± 35.93 ^c^
	1.0	1863.65 ± 13.49 ^c^	1367.66 ± 20.64 ^d^	2450.63 ± 41.92 ^b^	2378.21 ± 72.96 ^b^
	5.0	2708.21 ± 108.50 ^a^	1729.74 ± 74.63 ^b^	3904.41 ± 33.75 ^a^	2694.72 ± 58.82 ^a^
	10.0	2240.05 ± 195.17 ^b^	1244.03 ± 30.90 ^e^	1861.99 ± 12.24 ^c^	1821.16 ± 70.37 ^d^
Source of Variation		*p* > F			
Variety		<0.0001		<0.0001	
Water		<0.0001		<0.0001	
GB		<0.0001		<0.0001	
Variety × Water		<0.0001		<0.0001	
Variety × GB		<0.0001		<0.0001	
Water × GB		<0.0001		<0.0001	
Variety × Water × GB		<0.0001		<0.0001	

CK represents maize planted in normal field capacity; data are shown as the means ± standard deviation (*n* = 3, biological replicates); different letters in a water treatment indicate the least significant differences according to *p* value ≤ 0.05.

**Table 2:** The effect of glycinebetaine (GB) on leaf relative water content (g/g, %) of maize leaves after 8 d of water stress.

Water Treatments	GB (mM)	Heyu 397	Yindieyu 9
CK	0	95.39 ± 1.98 ^a^	97.12 ± 0.35 ^ab^
	0.5	97.48 ± 0.21 ^a^	96.59 ± 1.60 ^ab^
	1.0	96.80 ± 1.25 ^a^	98.35 ± 1.45 ^a^
	5.0	96.72 ± 1.59 ^a^	96.16 ± 0.79 ^b^
	10.0	96.71 ± 1.89 ^a^	95.65 ± 0.34 ^b^
Flooding	0	97.13 ± 0.67 ^ab^	95.84 ± 1.94 ^a^
	0.5	95.18 ± 1.80 ^b^	95.79 ± 0.59 ^a^
	1.0	97.44 ± 0.52 ^a^	96.35 ± 0.58 ^a^
	5.0	95.75 ± 0.41 ^ab^	97.23 ± 0.72 ^a^
	10.0	95.44 ± 1.43 ^ab^	97.19 ± 0.63 ^a^
Drought	0	68.04 ± 1.73 ^c^	71.50 ± 0.53 ^c^
	0.5	80.12 ± 1.64 ^b^	78.26 ± 2.66 ^bc^
	1.0	87.54 ± 0.69 ^a^	80.94 ± 0.57 ^ab^
	5.0	87.10 ± 4.45 ^a^	82.61 ± 2.78 ^a^
	10.0	79.76 ± 1.86 ^b^	82.04 ± 3.62 ^ab^
Source of Variation		*p* > F	
Variety		0.3565	
Water		<0.0001	
GB		<0.0001	
Variety × Water		0.0987	
Variety × GB		0.0188	
Water × GB		<0.0001	
Variety × Water × GB		0.0005	

CK represents maize planted in normal field capacity; data are shown as the mean ± standard deviation (*n* = 3, biological replicates); different letters in a water treatment indicate the least significant differences according to *p* value ≤ 0.05.

**Table 3:** The stepwise regression analysis for eight indicators with leaf dry matter (LDM).

Water Stress	Models	*R^2^*	*F*	*p > F*
Flooding	*LDM*_1_ = −0.858 + 0.071*X_APX_* + 0.014*X_PH_*	0.91	89.03	<0.001
Drought	*LDM*_2_ = −0.504 − 0.051*X_POD_* + 0.022*X_PH_*	0.80	32.35	<0.001

POD, APX, and PH represent guaiacol peroxidase, ascorbate peroxidase, and plant height, respectively.

**Table 4:** Analysis for tolerance after 8 d based on comprehensive evaluation (D) value and tolerance coefficient.

Varieties	Water Treatments	D Value	Tolerance Coefficient
Heyu 397	CK	0.53	-
	Flooding	0.10	0.82
	Drought	0.48	1.07
Yindieyu 9	CK	0.59	-
	Flooding	0.40	0.93
	Drought	0.50	1.12

CK represents maize planted in normal field capacity.

## Data Availability

The data presented in this study are available in the graphs and table provided in the manuscript.

## Abbreviations

*GB*	Glycinebetaine
*WS*	Water stress
*ROS*	Reactive oxygen species
*LA*	Leaf area
*SOD*	Superoxide dismutase
*POD*	Guaiacol peroxidase
*APX*	Ascorbate peroxidase
*V*3	Three-leaf stage
*V*5	Five-leaf stage
*H_*2*_O_*2*_*	Hydrogen peroxide
*O_*2*_^−^*	Superoxide anion
*RWC*	Relative water content
*CK*	Maize planted in normal field capacity
*FC*	Field capacity
*PBS*	Phosphate buffer solution
*OD*	Absorbance of the mixture
*FW*	Fresh weight
*D value*	Comprehensive evaluation value

## References

[1] (2022). EM-DAT. https://www.emdat.be/publications/.

[2] Wang B.C.H., Lian J., Peng Y., Hu B., Chen H. (2019). Generalized reference evapotranspiration models with limited climatic data based on random forest and gene expression programming in Guangxi, China. Agric. Water Manag..

[3] Wang G.Y., Ahmad S., Wang Y., Wang B.W., Huang J.H., Jahan M.S., Zhou X.B., Shi C.Q. (2023). Multivariate analysis compares and evaluates drought and flooding tolerances of maize germplasm. Plant Physiol..

[4] Ren B., Zhang J., Dong S., Zhao B. (2018). Responses of carbon metabolism and antioxidant system of summer maize to waterlogging at different stages. J. Agron. Crop Sci..

[5] Das R.R., Vinayan M.T., Seetharam K., Patel M., Phagna R.K., Singh S.B., Shahi J.P., Sarma A., Barua N.S., Babu R. (2021). Genetic gains with genomic versus phenotypic selection for drought and waterlogging tolerance in tropical maize (*Zea mays* L.). Crop J..

[6] Hasanuzzaman M., Bhuyan M.H.M.B., Zulfiqar F., Raza A., Mohsin S.M., Mahmud J.A., Fujita M., Fotopoulos V. (2022). Reactive oxygen species and antioxidant defense in plants under abiotic stress: Revisiting the crucial role of a universal defense regulator. Antioxidants.

[7] Ahmad S., Wang G.Y., Muhammad I., Chi Y.X., Zeeshan M., Nasar J., Zhou X.B. (2022). Interactive effects of melatonin and nitrogen improve drought tolerance of maize seedlings by regulating growth and physiochemical attributes. Antioxidants.

[8] Huang C., Zhang W., Wang H., Gao Y., Ma S., Qin A., Liu Z., Zhao B., Ning D., Zheng H. (2022). Effects of waterlogging at different stages on growth and ear quality of waxy maize. Agric. Water Manag..

[9] Barickman T.C., Simpson C.R., Sams C.E. (2019). Waterlogging causes early modification in the physiological performance, carotenoids, chlorophylls, proline, and soluble sugars of cucumber plants. Plants.

[10] Li B., Feng Y., Zong Y., Zhang D., Li P. (2020). Elevated CO_2_-induced changes in photosynthesis, antioxidant enzymes and signal transduction enzyme of soybean under drought stress. Plant Physiol. Bioch..

[11] Lukić N., Trifković T., Kojić D., Kukavica B. (2021). Modulations of the antioxidants defence system in two maize hybrids during flooding stress. J. Plant Res..

[12] Salah A., Nwafor C.C., Han Y., Liu L., Rashid M., Batool M., El-Badri A.M., Cao C., Zhan M. (2022). Spermidine and brassinosteroid regulate root anatomical structure, photosynthetic traits and antioxidant defense systems to alleviate waterlogging stress in maize seedlings. S. Afr. J. Bot..

[13] Azahar I., Ghosh S., Adhikari A., Adhikari S., Hossain Z. (2020). Comparative analysis of maize root sRNA transcriptome unveils the regulatory roles of miRNAs in submergence stress response mechanism. Environ. Exp. Bot..

[14] Qi M., Liu X., Li Y., Song H., Zhou G. (2021). Photosynthetic resistance and resilience under drought, flooding and rewatering in maize plants. Photosynth. Res..

[15] Wang X., Li X., Gu J., Shi W., Zhao H., Sun C., You S. (2023). Drought and waterlogging status and dominant meteorological factors affecting maize (*Zea mays* L.) in different growth and development stages in northeast China. Agronomy.

[16] Tian L.X., Bi W.S., Ren X.S., Li W.L., Li J. (2020). Flooding has more adverse effects on the stem structure and yield of spring maize (*Zea mays* L.) than waterlogging in northeast China. Eur. J. Agron..

[17] Tian L., Li J., Bi W., Zuo S., Li L., Li W., Sun L. (2019). Effects of waterlogging stress at different growth stages on the photosynthetic characteristics and grain yield of spring maize (*Zea mays* L.) under field conditions. Agric. Water Manag..

[18] Jia Y., Xiao W., Ye Y., Wang X., Wang Y. (2020). Response of photosynthetic performance to drought duration and re-watering in maize. Agronomy.

[19] Shemi R., Wang L., Zhang K., Gheith E.S.M.S. (2021). Effects of salicylic acid, zinc and glycine betaine on morphophysiological growth and yield of maize under drought stress. Sci. Rep..

[20] Sadaghiani F.M., Dehaghi M.A., Pirzad A., Fotokian M.H. (2019). Variation in yield and biochemical factors of German chamomile (*Matricaria recutita* L.) under foliar application of osmolytes and drought stress conditions. J. Herbmed. Pharmacol..

[21] Hamani A.K.M., Li S., Chen J., Amin A.S., Wang G., Xiaojun S., Zain M., Gao Y. (2021). Linking exogenous foliar application of glycine betaine and stomatal characteristics with salinity stress tolerance in cotton (*Gossypium hirsutum* L.) seedlings. BMC Plant Biol..

[22] Hernandez-Leon S.G., Valenzuela-Soto E.M. (2022). Glycine betaine is a phytohormone-like plant growth and development regulator under stress conditions. J. Plant Growth Regul..

[23] Nawaz M., Wang Z. (2020). Abscisic acid and glycine betaine mediated tolerance mechanisms under drought stress and recovery in *Axonopus compressus*, a new insight. Sci. Rep..

[24] Valenzuela-Soto E.M., Figueroa-Soto C.G., Kumar V., Burritt D.J., Fujita M., Mäkela P., Hossain M.A. (2019). Biosynthesis and degradation of glycine betaine and its potential to control plant growth and development. Osmoprotectant-Mediated Abiotic Stress Tolerance in Plants: Recent Advances and Future Perspectives.

[25] Elhakem A.H. (2019). Mitigation of the salinity influences on maize (*Zea mays* L.) productivity by exogenous applications of glycine betaine. J. Stress Physiol. Biochem..

[26] Ahmed N., Zhang Y., Li K., Zhou Y., Zhang M., Li Z. (2019). Exogenous application of glycine betaine improved water use efficiency in winter wheat (*Triticum aestivum* L.) via modulating photosynthetic efficiency and antioxidative capacity under conventional and limited irrigation conditions. Crop J..

[27] Rasheed R., Iqbal M., Ashraf M.A., Hussain I., Shafiq F., Yousaf A., Zaheer A. (2018). Glycine betaine counteracts the inhibitory effects of waterlogging on growth, photosynthetic pigments, oxidative defence system, nutrient composition, and fruit quality in tomato. J. Hortic. Sci. Biotech..

[28] Mäkelä P., Peltonen-Sainio P., Jokinen K., Pehu E., Setala H., Hinkkanen R., Somersalo S. (1996). Uptake and translocation of foliar-applied glycinebetaine in crop plants. Plant Sci..

[29] Hasanuzzaman M., Banerjee A., Bhuyan M.H.M.B., Roychoudhury A., Mahmud J.A., Fujita M. (2019). Targeting glycine betaine for abiotic stress tolerance in crop plants, physiological mechanism, molecular interaction and signaling. Phyton Int. J. Exp. Bot..

[30] Chen F., Fang P., Zeng W., Ding Y., Peng Y. (2020). Comparing transcriptome expression profiles to reveal the mechanisms of salt tolerance and exogenous glycine betaine mitigation in maize seedlings. PLoS ONE.

[31] Sharma J., Kumar S., Kumar N., Yadav N., Khyalia P., Sharma A. (2024). Evaluation of yield and quality attributes of barley cultivars with foliar spray of glycine betaine under lead toxicity. Cereal Res. Commun..

[32] Yang X.H., Lu C.M. (2006). Effects of exogenous glycinebetaine on growth, CO_2_ assimilation, and photosystem II photochemistry of maize plants. Physiol. Plantarum.

[33] Huang Y., Li J., Duan L., Li Z. (2011). Drought resistance of maize seedlings induced by betaine. J. Maize Sci..

[34] Yang X., Song T., Liu H., Wang H. (2017). Effects of exogenous glycine betaine on the growth and chlorophyll content of maize seedlings under NaCl stress. Hubei Agr. Sci..

[35] Lukatkin A.S. (2002). Contribution of oxidative stress to the development of cold-induced damage to leaves of chilling-sensitive plants: Reactive oxygen species formation during plant chilling. Russ. J. Plant Physiol..

[36] Velikova V., Yordanov I., Edreva A. (2000). Oxidative stress and some antioxidant systems in acid rain-treated bean plants. Plant Sci..

[37] Giannopolitis C.N., Ries S.K. (1977). Superoxide dismutases: I. occurrence in higher plants. Plant Physiol..

[38] Cakmak I., Marschner H. (1992). Magnesium deficiency and high light intensity enhance activities of superoxide dismutase ascorbate peroxidase and glutathione reductase in bean leaves. Plant Physiol..

[39] Nakano Y., Asada K. (1987). Purification of ascorbate peroxidase in spinach chloroplasts; its inactivation in ascorbate-depleted medium and reactivation by monodehydroascorbate radical. Plant Cell Physiol..

[40] Bates L.S., Waldren R.P., Teare I.D. (1973). Rapid determination of free proline for water-stress studies. Plant Soil.

[41] Ahmad S., Wang G.Y., Muhammad I., Farooq S., Kamran M., Ahmad I., Zeeshan M., Huang J.H., Zhou X.B. (2022). Application of melatonin-mediated modulation of drought tolerance by regulating photosynthetic efficiency, chloroplast ultrastructure, and endogenous hormones in maize. Chem. Biol. Technol. Agric..

[42] Zou J., Hu W., Li Y., He J., Zhu H., Zhou Z. (2020). Screening of drought resistance indices and evaluation of drought resistance in cotton (*Gossypium hirsutum* L.). J. Integr. Agric..

[43] Fang W., Huang S., Huang G., Huang Q., Wang H., Wang L., Zhang Y., Li P., Ma L. (2019). Copulas-based risk analysis for inter-seasonal combinations of wet and dry conditions under a changing climate. Int. J. Climatol..

[44] Bi W., Weng B., Yan D., Wang H., Wang M., Yan S., Jing L., Liu T., Chang W. (2022). Responses of phosphate-solubilizing microorganisms mediated phosphorus cycling to drought-flood abrupt alternation in summer maize field soil. Front. Microbiol..

[45] Prazeres C.S., Coelho C.M.M., Souza C.A. (2021). Biochemical compounds and enzymatic systems related to tolerance to water deficit of maize seedlings. Plant Physiol. Rep..

[46] Salah A., Ming Z., Cao C.G., Han Y.L., Ling L., Liu Z.H., Li P., Ye M., Jiang Y. (2019). γ-aminobutyric acid promotes chloroplast ultrastructure antioxidant capacity and growth of waterlogged maize seedlings. Sci. Rep..

[47] Sarkar B., Bandyopadhyay P., Das A., Pal S., Hasanuzzaman M., Adak M.K. (2023). Abscisic acid priming confers salt tolerance in maize seedlings by modulating osmotic adjustment, bond energies, ROS homeostasis, and organic acid metabolism. Plant Physiol. Bioch..

[48] Zhang Y., Liu G., Dong H., Li C. (2020). Waterlogging stress in cotton: Damage, adaptability, alleviation strategies, and mechanisms. Crop J..

[49] Hasanuzzaman M., Ahmed N., Saha T., Rahman M., Rahman K., Alam M.M., Rohman M.M. (2022). Exogenous salicylic acid and kinetin modulate reactive oxygen species metabolism and glyoxalase system to confer waterlogging stress tolerance in soybean (*Glycine max* L.). Plant Stress.

[50] Ren B., Yu W., Liu P., Zhao B., Zhang J. (2022). Responses of photosynthetic characteristics and leaf senescence in summer maize to simultaneous stresses of waterlogging and shading. Crop J..

[51] Xiong Q., Cao C., Shen T., Zhong L., He H.H., Chen X. (2019). Comprehensive metabolomic and proteomic analysis in biochemical metabolic pathways of rice spikes under drought and submergence stress. Biochim.Biophys. Acta Proteins Proteom..

[52] Jinsheng Y., Wenjie G., Jiwang Z., Baizhao R., Lichun W. (2022). Responses of the lodging resistance of summer maize with different gene types to plant density. Agronomy.

[53] Li H., Li Z., Shen Z.J., Luo M.R., Zheng H.L. (2020). Physiological and proteomic responses of mangrove plant *Avicennia marina* seedlings to simulated periodical inundation. Plant Soil.

[54] Mcgee T., Shahid M.A., Beckman T., Chaparro J.X., Sarkhosh A. (2021). Physiological and biochemical characterization of six prunus rootstocks in response to flooding. Environ. Exp. Bot..

[55] Anjum S.A., Ashraf U., Tanveer M., Khan I., Hussain S., Shahzad B., Zohaib A., Abbas F., Saleem M.F., Ali I. (2017). Drought induced changes in growth, osmolyte accumulation and antioxidant metabolism of three maize hybrids. Front. Plant Sci..

[56] Sun F.L., Chen Q., Chen Q.J., Jiang M., Qu Y.Y. (2021). Screening of key drought tolerance indices for cotton at the flowering and boll setting stage using the dimension reduction method. Front. Plant Sci..

[57] Zhang K., Lyu W., Gao Y., Zhang X., Sun Y., Huang B. (2021). Choline-mediated lipid reprogramming as a dominant salt tolerance mechanism in grass species lacking glycine betaine. Plant Cell Physiol..

[58] Qi X., Li Q., Ma X., Qian C., Wang H., Ren N., Shen C., Huang S., Xu X., Xu Q. (2019). Waterlogging-induced adventitious root formation in cucumber is regulated by ethylene and auxin through reactive oxygen species signalling. Plant Cell Environ..

